# Direct inference of Patlak parametric images in whole-body PET/CT imaging using convolutional neural networks

**DOI:** 10.1007/s00259-022-05867-w

**Published:** 2022-06-18

**Authors:** Neda Zaker, Kamal Haddad, Reza Faghihi, Hossein Arabi, Habib Zaidi

**Affiliations:** 1 Division of Nuclear Medicine and Molecular Imaging, Department of Medical Imaging, Geneva University Hospital, CH-1211 Geneva 4, Switzerland; 2 School of Mechanical Engineering, Department of Nuclear Engineering, Shiraz University, Shiraz, Iran; 3 Geneva University Neurocenter, Geneva University, Geneva, Switzerland; 4 Department of Nuclear Medicine and Molecular Imaging, University of Groningen, University Medical Center Groningen, Groningen, Netherlands; 5 Department of Nuclear Medicine, University of Southern Denmark, Odense, Denmark

**Keywords:** Dynamic PET imaging, Clinical oncology, Deep learning, Patlak analysis, Lesion detectability

## Abstract

**Purpose:**

This study proposed and investigated the feasibility of estimating Patlak-derived influx rate constant (*K*_*i*_) from standardized uptake value (SUV) and/or dynamic PET image series.

**Methods:**

Whole-body ^18^F-FDG dynamic PET images of 19 subjects consisting of 13 frames or passes were employed for training a residual deep learning model with SUV and/or dynamic series as input and *K*_*i*_-Patlak (slope) images as output. The training and evaluation were performed using a nine-fold cross-validation scheme. Owing to the availability of SUV images acquired 60 min post-injection (20 min total acquisition time), the data sets used for the training of the models were split into two groups: “With SUV” and “Without SUV.” For “With SUV” group, the model was first trained using only SUV images and then the passes (starting from pass 13, the last pass, to pass 9) were added to the training of the model (one pass each time). For this group, 6 models were developed with input data consisting of SUV, SUV plus pass 13, SUV plus passes 13 and 12, SUV plus passes 13 to 11, SUV plus passes 13 to 10, and SUV plus passes 13 to 9. For the “Without SUV” group, the same trend was followed, but without using the SUV images (5 models were developed with input data of passes 13 to 9). For model performance evaluation, the mean absolute error (MAE), mean error (ME), mean relative absolute error (MRAE%), relative error (RE%), mean squared error (MSE), root mean squared error (RMSE), peak signal-to-noise ratio (PSNR), and structural similarity index (SSIM) were calculated between the predicted *K*_*i*_-Patlak images by the two groups and the reference *K*_*i*_-Patlak images generated through Patlak analysis using the whole acquired data sets. For specific evaluation of the method, regions of interest (ROIs) were drawn on representative organs, including the lung, liver, brain, and heart and around the identified malignant lesions.

**Results:**

The MRAE%, RE%, PSNR, and SSIM indices across all patients were estimated as 7.45 ± 0.94%, 4.54 ± 2.93%, 46.89 ± 2.93, and 1.00 ± 6.7 × 10^−7^, respectively, for models predicted using SUV plus passes 13 to 9 as input. The predicted parameters using passes 13 to 11 as input exhibited almost similar results compared to the predicted models using SUV plus passes 13 to 9 as input. Yet, the bias was continuously reduced by adding passes until pass 11, after which the magnitude of error reduction was negligible. Hence, the predicted model with SUV plus passes 13 to 9 had the lowest quantification bias. Lesions invisible in one or both of SUV and *K*_*i*_-Patlak images appeared similarly through visual inspection in the predicted images with tolerable bias.

**Conclusion:**

This study concluded the feasibility of direct deep learning-based approach to estimate *K*_i_-Patlak parametric maps without requiring the input function and with a fewer number of passes. This would lead to shorter acquisition times for WB dynamic imaging with acceptable bias and comparable lesion detectability performance.

**Supplementary Information:**

The online version contains supplementary material available at 10.1007/s00259-022-05867-w.

## Introduction

Positron emission tomography (PET) is a well-established imaging modality in clinical oncology for diagnostics, staging, monitoring of treatment response, and radiation treatment planning. PET with glucose analog 2-deoxy-2-[^18^F] fluoro-D-glucose (^18^F FDG) tracer demonstrated its capability in diagnosing infections, inflammation, and a variety of malignancies [1]. Currently, static PET imaging, where multiple bed positions at late time points, after reaching the equilibrium, are acquired during a single time frame, is the most commonly used method in the clinic. Semi-quantitative image-derived PET metrics, such as the standardized uptake value (SUV), supports the physician’s qualitative interpretation [2]. Static PET imaging does not take advantage of the full potential of PET since the tracer distribution is fundamentally a dynamic process that can be acquired using dynamic imaging protocols [3]. Patlak [4], spectral analysis [5], and the more complex full compartmental modeling method [6] are among the strategies used for the generation of parametric maps. Since the standard Patlak model is a fast linear graphical analysis technique, it is a suitable for macro-parameter estimation at the voxel level [3]. Unlike the SUV semi-quantitative index, quantitative parameters of glucose uptake rates take into account the plasma FDG dynamics [7]. Another advantage of Patlak graphical analysis is its power in dynamic whole-body PET imaging [8, 9]. This technique requires only voxelwise time-activity curve (TAC) measurements after kinetic equilibrium is achieved, and hence does not require complete scanning of all beds. Whole-body scanning starting from the injection time is not feasible owing to the limited axial FOV of most commercial PET scanners used in the clinic. Moreover, Patlak graphical analysis reduces the time-consuming non-linear estimation of the kinetic micro-parameters to linear regression to determine the macro-parameters. The simple Patlak method approximates a formula that arranges the [10] measured time-activity curves in steady-state as a weighted sum of the input function and it is integral. These weights are called Patlak slope and intercept. The Patlak slope can represent the net transfer rate or influx constant [11].

One of the reasons preventing whole-body (WB) dynamic PET imaging from being routinely employed in clinical setting is the difficulty associated with the estimation of the input function (IF). Invasive arterial or venous blood sampling is the common approach for estimating the IF, although image-derived IF is an alternative approach commonly used in dynamic PET imaging protocols [12–14]. The IF is usually sampled from the left ventricle or atrium [15], although using the ascending or abdominal aorta was also suggested as an alternative approach [16]. The partial volume effect is an issue for small blood pools and also for the left ventricle or atrium owing to the high contrast between the cavities and the myocardium and hence the larger impact of partial volume effect [3]. In this light, extracting the input function from images suffers from a number of limitations impacting the accuracy of the approach. Moreover, estimating the IF from the blood samples is an invasive procedure that is logistically difficult to implement in clinical setting. It should be noted that due to the existence of radioactive metabolites in the blood for many radiotracers, image-derived IF estimation is challenging [3]. Another difficulty of dynamic WB PET imaging is its long acquisition time. Although various groups attempted to reduce the acquisition time through selecting a fraction of the range of time windows, from 45–60 min post-injection [10, 17] to about 0–100 min post-injection [18] in dynamic whole-body protocols, the acquisition time still remains to be optimized. As demonstrated in [16, 19–25], the acquisition time is too long for patients to tolerate and hence motion artifacts might be inevitable. Moreover, these protocols tend to reduce patients’ throughput.

Application of artificial intelligence in nuclear medicine has been extensively discussed [26–29]. Smith et al. [30] developed a model-free reinforcement learning (RL) algorithm, referred to as Q learning with a novel reward function to detect instances of an object in PET images. They concluded that the RL framework is promising for automated object detection from PET images. Moreover, Ackerley et al. [31] demonstrated that using a set of CNNs trained on PET data with a simple patch-based approach, blinded to anatomical locations during training and classification, would result in an efficient decision support tool for automated detection and segmentation of malignant uptakes. In another study by Feng et al. [32], they trained a CNN model to predict direct parametric image reconstructions generated from list-mode or sinogram data (direct method) from parametric images generated using image-based analysis after dynamic image reconstruction (indirect method).

Deep learning techniques demonstrated promising results for approximating four perfusion parameters without performing an explicit deconvolution method [33]. Das et al. [34] employed a simple effective method utilizing random forest regression for multi-parameter estimation in MR spectroscopic imaging. Hence, by using machine learning techniques, such as random forest-based regression, metabolite quantification can be performed faster. A novel deep learning-based approach for direct estimation of the PK parameters from under-sampled DCE-MRI data was also proposed [35]. Zou et al. [36] proposed a method to estimate the pharmacokinetic (PK) parameters by extracting long and short time-dependent features in DCE-MRI. Using this method, the inference time could be reduced because of the small computational burden of the long short-term memory (LSTM). Moreover, they indicated that the LSTM was much more robust to the temporally subsampled DCE data than the direct PK model fitting. The computation time acceleration was approximately 90-folds compared with the direct PK model fitting approach. Therefore, it can be concluded that machine learning approaches including RNN and CNN networks could be useful in estimating PK parameters in comparable shorter times.

A related study was conducted by Ulas et al. [37] wherein they predicted pharmacokinetic parameters based on Patlak or eTofts model directly by feeding time series of dynamic contrast-enhanced (DCE)-MRI data into a neural network. Their results demonstrated that their model could accurately generalize to new cases even if the specific arterial input function (AIF) of the input subjects is not available. They evaluated their approach on a brain data set that exhibited a shorter processing time compared to convolutional non-linear least-squares fitting. The proposed deep learning-based solution in this work does not require the input function while offering a very short computational time. They also demonstrated that although the standard Patlak and eTofts model can fit the data better compared to the CNN model trained with these models separately, the difference is not significant, which shows that CNN model could achieve high accuracy with less than 2% fitting error on average. Moreover, for the CNN model trained on Patlak model parameters, tissue types, such as the white matter and gray matter, could be differentiated successfully by *K*^trans^ parameter. In addition to that, they indicated that the localized smoother areas can be produced by the CNN model in regions with discontinuities of parameter values arising especially at highly perfused regions, such as the vessels. They concluded that the proposed ML model can be used as an appropriate parameter inference model for quantification of subtle blood–brain barrier (BBB) permeability, which is vital in the diagnosis of several diseases, such as cerebral small vessel disease, lacunar stroke, and vascular dementia. To address the above-mentioned challenges of dynamic PET imaging, we propose a direct and fast method for generating Patlak maps from dynamic passes and/or SUV images with the aid of deep learning techniques.

For training the deep learning model, combinations of passes and/or SUV images were used as the input data sets. SUV images are commonly acquired in clinical routine, and given the longer acquisition time (20 min compared to 3 min for dynamic passes), they inherently bear higher signal-to-noise ratio. Therefore, the impact of employing SUV images on the deep learning-based prediction of parametric images was studied through splitting the data set into “With SUV” and “Without SUV.” Moreover, to determine the adequate number of passes for generating accurate Patlak images with the aim to minimize the error between the reference and predicted images, passes starting from pass 13 (the last pass in our protocol) were added one by one to the models (either with or without SUV images). The models were evaluated through different statistical metrics for finding as few numbers of passes as possible. No input function estimation was used for the training of the deep learning models. The *K*_*i*_ images were produced from the image-derived input function and an irreversible two-tissue compartment model was considered reference for evaluating our models. In addition to the voxel-based whole-body evaluation, we compared lesion detectability performance between the predicted and reference images.

## Materials and methods

### Data sets and data acquisition

#### Patient population

Nineteen patients referred for staging and restaging of lung or abdominal lesions through ^18^F-FDG PET/CT examinations performed were included in this study. The study was approved by the local ethics committee and written informed consent was obtained. The average age of the patients (7 females and 12 males) was 59.79 ± 10.38 years.

#### PET/CT data acquisition

PET data acquisition on a Siemens Biograph™ mCT scanner started after injecting an activity of 3.71 ± 1.05 MBq/Kg of ^18^F-FDG. The scan duration time was about 80 min to acquire sequential dynamic and static scans. A low-dose CT scan (120 kVp and 80 mAs) was acquired for attenuation correction. The first acquisition performed post-injection was a 6-min dynamic single-bed acquisition in the blood pool region to estimate the IF. List-mode data of this bed scan were split into 20 frames (8 × 5 s, 4 × 10 s, 4 × 25 s, 4 × 45 s). In the next step, time-of-flight dynamic WB scans (head-to-thigh) in continuous bed motion (CBM) mode at a fixed bed speed of 4 mm/s were acquired. Finally, for comparison with the static PET acquisition protocol, an SUV WB CBM scan of about 20 min starting at ~ 60 min post-injection was also acquired. Contrast-enhanced CT images of 15 out of 19 patients were acquired for diagnostic purposes. The images were reconstructed using 3D iterative ordinary Poisson-ordered subset expectation–maximization algorithm with resolution recovery (2 iterations and 21 subsets). A Gaussian filter of 2 mm FWHM was applied post-reconstruction.

### Methodology for whole-body parametric imaging

Patlak graphical analysis is the method of choice for this dynamic protocol since it does not require scans to sample the early tracer kinetics and also it relies on a simple linear fit to obtain the slope and intercept parameters [3]. It should be mentioned that the linear fit is reasonable as long as the PET scans are acquired when a relative equilibrium is reached between the reversible and vascular tissue compartments which is obtained 5 to 10 min after ^18^F-FDG injection [3].

1 $$\frac{C(t)}{{C}_{p}(t)}= {K}_{i} \frac{{\int }_{0}^{t}{C}_{p}\left(\tau \right)d\tau }{{C}_{p}(t)}+V$$

*C(t)* is the activity concentration, *C*_*p*_*(t)* is the blood plasma time/activity concentration over time or plasma input function (PIF), *K*_*i*_ (Patlak slope) is the uptake rate constant or net influx, and *V* (Patlak intercept) is referred to as the distribution volume. This formula is applied voxelwise and as such, Patlak parametric images of slope and intercept are produced. In this work, we applied deep learning techniques to predict Patlak slope to take advantage of its complementary role to static PET imaging for the task of lesion detection [8, 9].

### Network architecture and training of the network

A ResNet model [38] was employed for direct prediction of *K*_*i*_-Patlak maps from SUV and/or passes images without using an input function. The whole model training and evaluation process was implemented using the NiftyNet platform [39]. NiftyNet is an open-source Tensorflow-based (CNNs) platform with specific functionality for medical image analysis, which provides tools for image segmentation, regression, generation, and representation learning. Overall, the NiftyNet platform facilitates efficient deep learning-assisted medical image analysis and also reduces duplication of effort in the field.

**Fig. 1:**
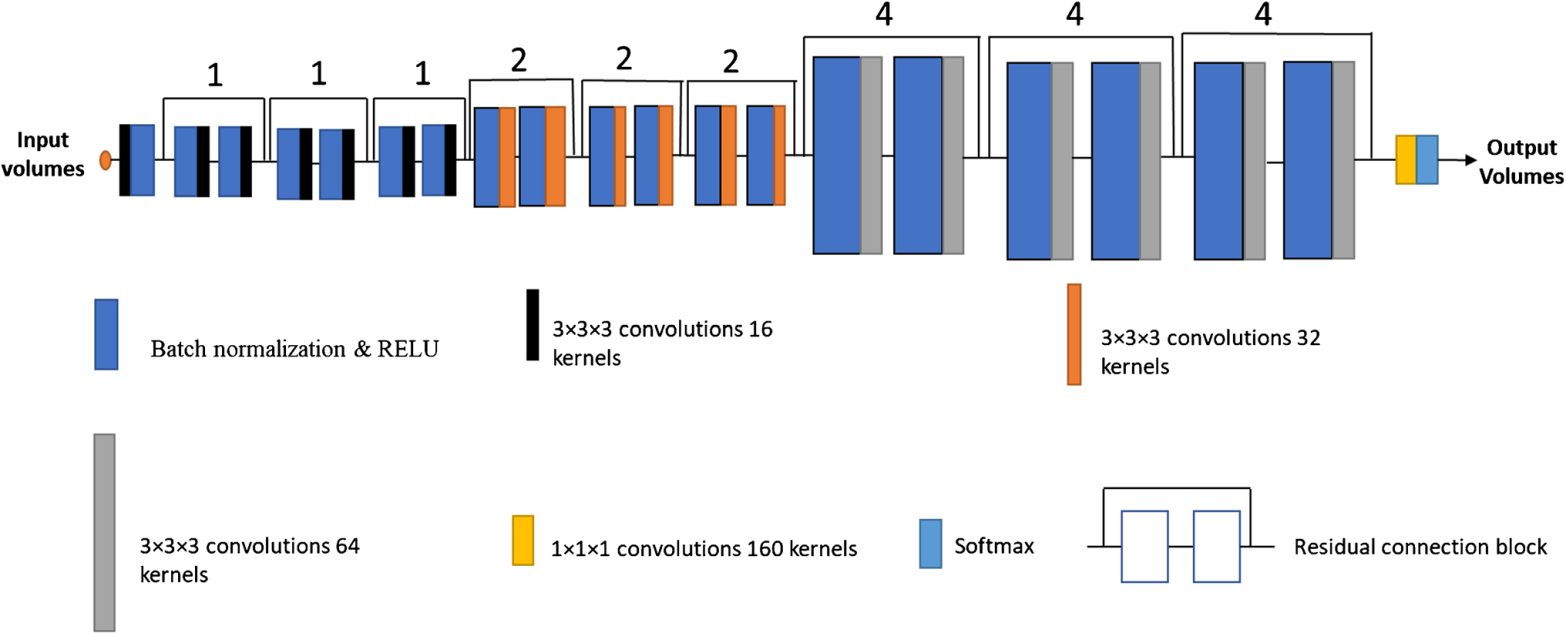
Sketch of the highres3Dnet neural network architecture. The numbers above the residual connection blocks exhibit the dilation factors [38]

The ResNet architecture [38, 39], as exhibited in Fig. 1, consists of 20 convolutional layers. Each residual block consists of a convolutional layer, a batch normalization layer, and an element-wise rectified linear unit (ReLU), which are arranged in the pre-activation order [40]. Low-level image features, such as edges and corners, are captured by the first 7 layers, which were not dilated. The implemented convolutions are 3 × 3 × 3-voxel convolutions. The next six convolutional layers are dilated by a factor of 2 and the last 6 residual convolutional layers are dilated by 4. The batch normalization layer associated with each convolutional layer is mainly used for training convergence [41]. ReLU’s role is to add non-linearity for improving the network ability to extract the discriminative features [42]. The main purpose of applying dilated convolution is to replace the pooling operations (2-stride convolutions) in the original ResNet [43] for increasing the resolution of the network’s output [44].

### Data preparation and training

Prior to training, all PET images and parametric images were preprocessed/normalized. The input data sets were categorized into two groups: “with SUV” and “without SUV.” In the “with SUV” group, the input data sets included SUV images alone for training the first model and added a pass each time for training other models starting from the last pass (i.e., SUV plus pass 13, SUV plus passes 13 and 12, SUV plus passes 13 to 11, SUV plus passes 13 to 10, and SUV plus passes 13–9 (6 input data sets)) to evaluate the impact of SUV images on the model’s prediction. In the “without SUV” group, we followed the same trend but without SUV images (starting directly from the last pass (pass 13) and added one pass (12, 11, 10, 9 …) each time to investigate the effect of the number of passes on the accuracy of the prediction model. The output data set involved reference *K*_*i*_-Patlak images generated using our in-house developed code [22]. To generate Patlak parameters using our in-house developed code, an image-derived IF is required. The code uses a modified hybrid whole-body dynamic protocol that allows the estimation of micro-parameters in the initial bed targeting the blood pool in addition to calculating whole-body macro-parameter maps. In this new protocol, in order to avoid restricting the initial bed position to the heart region, it was proposed to choose the region for input function (IF) extraction based on the location of the suspected pathology and from the descending or ascending aorta. Therefore, in this work, depending on the location of the primary malignancy and therefore the site of the initial bed position, the IF can be extracted from the heart region or aorta. Overall, 11 independent deep learning models were developed. The input data sets including SUV and passes were converted to SUV units to reduce the dynamic range of image intensities. All images were normalized within the range [0–2]. Therefore, the maximum value of each data set was calculated and then the maximum values averaged over the 19 patients and the averaged number divided by 2. To maintain the quantitative value of PET and parametric images, fixed normalization factors were employed for all subjects and different types of data. A fixed normalization factor was determined for each type of image data. To normalize each type of images to the range [0–2], the average of the maximum values was selected as the normalization factor. No intensity cropping/clipping was performed on the normalized images. Hence, images of the different subjects may have different maximum values within the range of 0 to almost 2. Thereafter, SUV images and dynamic frames were scaled accordingly. Subsequently, all images were cropped to a matrix size of 168 × 168 voxels to reduce the computational cost through eliminating the irrelevant background pixels in the images.

The training of the models was performed using a nine-fold cross-validation scheme. At each iteration, 2 patients were kept as an external test (at the last iteration 3 patients were kept out). In this regard, all subjects were excluded once as the validation data set. The training was performed in two-dimensional mode using a 168 × 168 window size (each pair of transaxial slices of the input and output data were considered as a single training sample). Two-dimensional implementation of the deep learning model is beneficial when the number of training samples is not sufficiently large for three-dimensional implementation, wherein each two-dimensional slice is regarded as a training sample. In this light, we sought to implement the models in two-dimensional mode since 19 training subjects were not sufficient for a three-dimensional implementation. The selected parameters for model training were as follows: learning rate = 0.003–0.0001, optimizer = adam, loss function = L2Loss, decay = 0.00001–0.0000, batch_size = 20, and sample per volume = 1. Five percent of the training data set was considered for model evaluation during training to avoid the risk of overfitting. No overfitting was observed in monitoring the differences between the evaluation and training losses (errors). Ten epochs of training led to a plateau of the training loss. The learning curves of all nine-fold of validations are depicted in the supplementary section (Supplementary Figs. 2–10).

### Evaluation strategies

#### Voxel-based assessment

The performance of the trained models in the image domain was evaluated through comparing the deep learning-based synthesized *K*_*i*_-Patlak images with their reference counterparts. The negative-valued or less than 5 × 10^−5^ voxel values were excluded from evaluation after converting back the voxel values to the original intensity ranges. Only few pixels located mostly in the background of some images had negative values. The predicted *K*_*i*_-Patlak images of the 11 models were evaluated using whole-body images and voxelwise metrics, including the mean absolute error (MAE) (Eq. 2), mean error (ME) (Eq. 3), mean relative absolute error (MRAE%) (Eq. 4), relative error (RE%) (Eq. 5), root mean square error (RMSE) (Eq. 6), mean square error (MSE) (Eq. 7), peak signal-to-noise ratio (PSNR) (Eq. 8), and structural similarity index (SSIM) (Eq. 9).

2 $$MAE= \frac{1}{N} \sum\nolimits_{i=1}^{N}\left|\left({K}_{{i}_{predict}}\left(i\right)- {K}_{{i}_{ref}}\left(i\right)\right)\right|$$

3 $$ME= \frac{1}{N} \sum\nolimits_{i=1}^{N}\left({K}_{{i}_{predict}}\left(i\right)-{K}_{{i}_{ref}}\left(i\right)\right)$$

4 $$MRAE\%= \left(\frac{1}{N}\sum\nolimits_{i=1}^{N}\left|\frac{{K}_{{i}_{predict}}\left(i\right)-{K}_{{i}_{ref}}\left(i\right)}{{K}_{{i}_{ref}}\left(i\right)}\right|\right)\times 100$$

5 $$RE\%= \left(\frac{1}{N}\sum\nolimits_{i=1}^{N}\frac{{K}_{{i}_{predict}}\left(i\right)-{K}_{{i}_{ref}}\left(i\right)}{{K}_{{i}_{ref}}\left(i\right)}\right) \times 100$$

6 $$RMSE= \sqrt{\frac{1}{N}\sum\nolimits_{i=1}^{N}{\left({K}_{{i}_{predict}}\left(i\right)-{K}_{{i}_{ref}}\left(i\right)\right)}^{2}}$$

7 $$MSE= \frac{1}{N} \sum\nolimits_{i=1}^{N}{\left({K}_{{i}_{predict}}\left(i\right)-{K}_{{i}_{ref}}\left(i\right)\right)}^{2}$$

8 $$PSNR=10\;\log\frac{I^2}{MSE}$$

9 $$SSIM= \frac{\left(2{\mu }_{ref}{\mu }_{predict}+{K}_{1}\right)\left(2{\delta }_{ref,predict}\right)}{\left({{\delta }^{2}}_{ref}+{{\mu }^{2}}_{predict}+{K}_{1}\right)\left({{\delta }^{2}}_{ref}+{{\delta }^{2}}_{predict}+{K}_{2}\right)}$$

*N* is the total number of voxels of the ground truth image and *i* represents the corresponding *i*^*th*^ voxel in the synthesized and ground truth images. *I* stands for the maximum intensity values of the predicted and reference images and MSE is the mean square error. *µ*_ref_ and *µ*_predict_ are the mean values of reference and predicted *K*_*i*_-Patlak images, respectively. *δ*_ref_ and *δ*_predict_ stand for the variances of reference and predicted *K*_*i*_-Patlak images, whereas *δ*_ref,predict_ represents the covariance. The parameters *K*_1_ = (*K*_1_I)^2^ and *K*_2_ = (*K*_2_I)^2^ with the constants *K*_1_ = 0.01 and *K*_2_ = 0.02 were considered for preventing the division by very small values. To compute these formulas, each index was calculated for each patient, and hence, *N* is the number of voxels for each subject. The average value for all subjects was considered the mean value for that index. In fact, these indices were calculated patient-wise (as opposed to slice-wise). Since whole-body image analysis metrics may not be adequate for the assessment of the models, the quantitative metrics were also calculated for specific regions/organs, including the heart, lung, liver, and brain as well as lesions/hot spots.

### Organ and lesion-based evaluations

Ten-millimeter-diameter spherical volumes of interest (VOIs) were drawn within organs, such as the brain, lung, liver, and heart. The absolute mean error (AME) (Eq. 10) and absolute mean relative error (AMRE%) (Eq. 11) were calculated for each organ and for each model with different inputs.

10 $$AME= \left|\frac{1}{N}\sum\nolimits_{i=1}^{N}\left({K}_{{i}_{predict}}\left(i\right)-{K}_{{i}_{ref}}\left(i\right)\right)\right|$$

11 $$AMRE\%= \left|\frac{\sum_{i=1}^{N}{K}_{{i}_{predict}}-\sum_{i=1}^{N}{K}_{{i}_{ref}}}{\sum_{i=1}^{N}{K}_{{i}_{ref}}}\right|$$

To investigate the ability of our models to detect and localize lesions and other pathologies, the findings in all case studies were depicted and analyzed. The AME, AMRE, tumor-to-background ratio (TBR) (Eq. 12), and contrast-to-noise ratio (CNR) (Eq. 13) were among the calculated metrics.

12 $$\mathrm{TBR }= \left({\mathrm{Tumor ROI}}_{\mathrm{max}}/{\mathrm{Background ROI}}_{\mathrm{mean}}\right)-1$$

13 $$\mathrm{CNR}=\mathrm{TBR}/\mathrm{Background}\;{\mathrm{ROI}}_{\mathrm{SD}}$$

## Results

### Whole-body comparison of pharmacokinetic maps

**Fig. 2:**
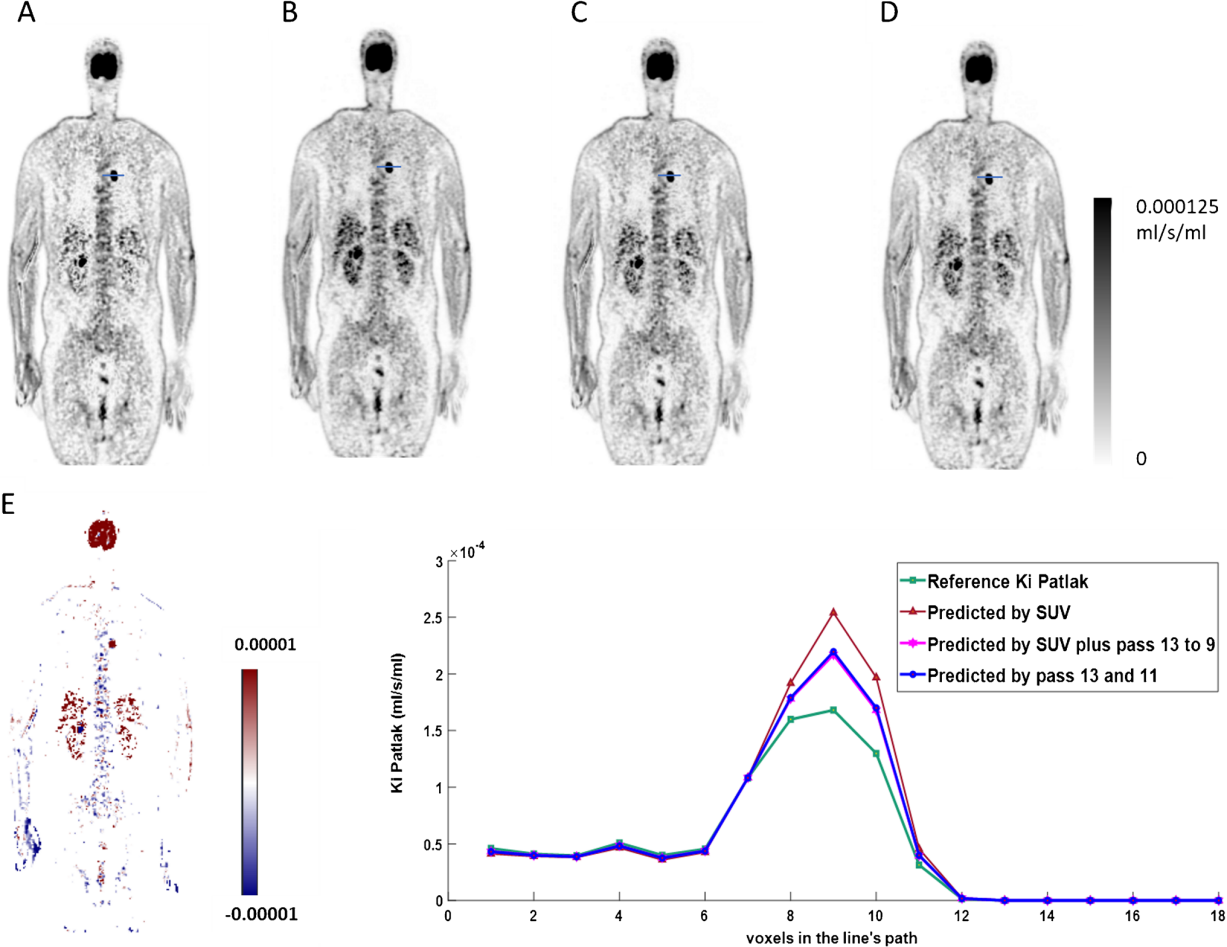
Representative coronal views showing **A** reference *K*_*i*_-Patlak, **B** predicted *K*_*i*_-Patlak by SUV input, **C** predicted *K*_*i*_-Patlak by SUV plus passes 13–9, **D** predicted *K*_*i*_-Patlak by passes 13–11, **E** the difference bias map between *K*_i_ predicted by SUV plus passes 13 to 9 input—reference *K*_i_, **F** the horizontal line profile drawn through the hypermetabolic lung nodule on the reference and the predicted *K*_i_ maps when using different inputs

Figure 2 exhibits coronal views of the reference and the predicted *K*_*i*_-Patlak maps generated using only SUV, SUV plus passes 13 to 9, and passes 13 to 11 as input. The visual inspection revealed no remarkable differences between the predicted *K*_*i*_ images when using different inputs. A horizontal line profile was drawn through a hypermetabolic nodule at the level of the apical segment of the lower lung left lobe (SUV_max_ = 14.2). The line profile depicted insignificant differences between the reference and predicted parametric images. The SUV alone model resulted in the largest error, whereas the line profiles of the two other models trained with SUV plus passes 13–9 and passes 13–11 exhibited minor differences. Moreover, the bias map calculated between reference and predicted *K*_*i*_ maps by SUV plus passes 13–9 as input data set (Fig. 2E) shows over-/underestimation of the slope or influx rate parameter.

**Fig. 3:**
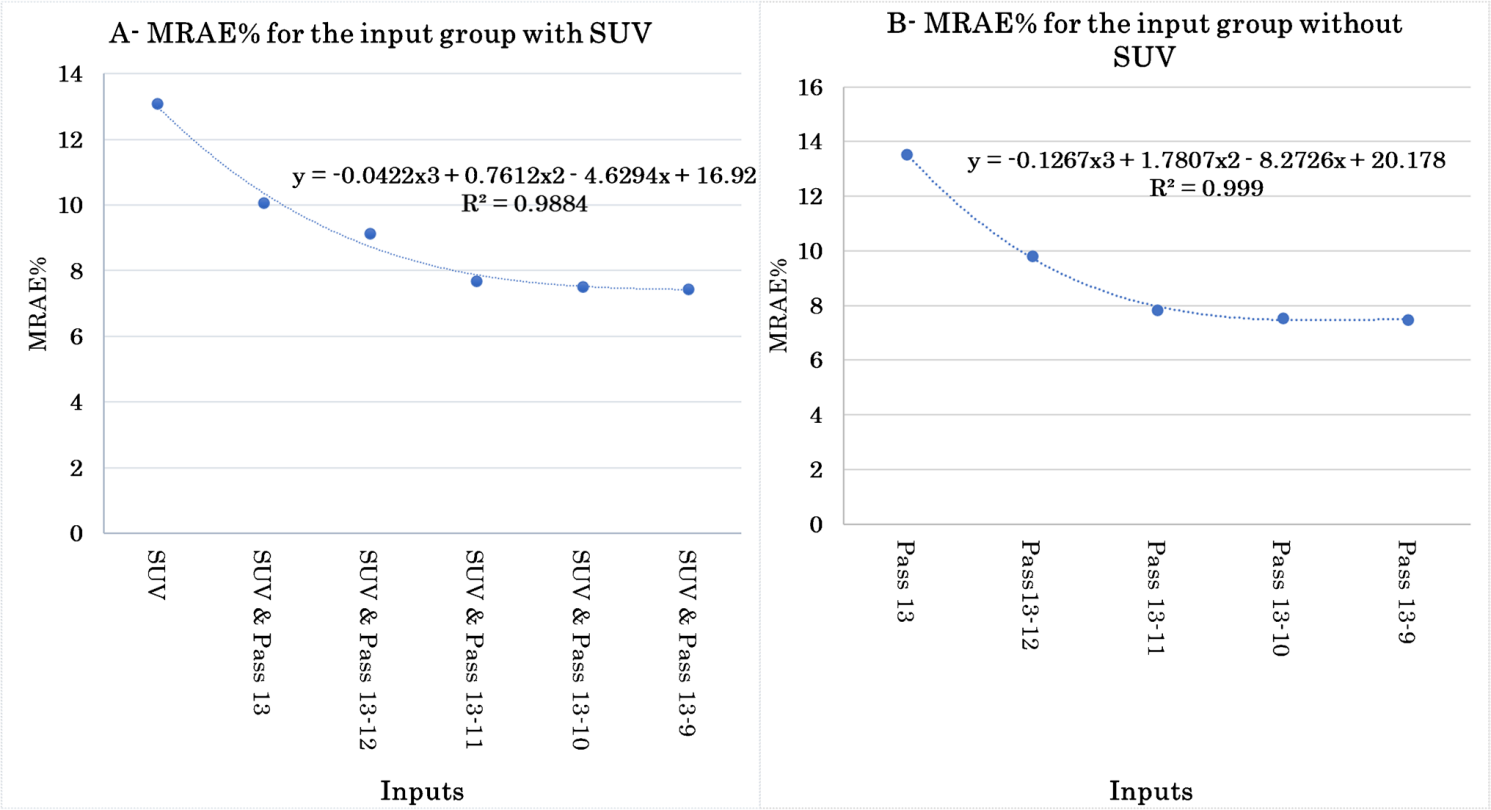
The MRAE% versus different inputs for groups: **A** “with SUV” and **B** “without SUV”

Figure 3 depicts the MRAE% error for both groups (“with SUV” and “without SUV” as input data sets) against their subsequent members. Each point of this graph is the average voxel-based MRAE for all study cases. It was observed that by adding a pass to the input data set, the mean relative absolute error decreases and after pass 11. Adding more passes to the input data set would not result in a significant improvement in model performance. Moreover, both groups exhibited the same trend in terms of estimating the parametric images, except that the “with SUV” group exhibited slightly lower errors compared to the “without SUV” group. The same trend was observed for other metrics, including MAE, ME, RE%, RMSE, and MSE. To illustrate the trend of error reduction, the same graphs were sketched for organs, such as the brain, lung, heart, and liver, wherein the same trend was observed.

In spite of the promising results achieved using the deep learning models, there was a single case (patient # 3) with unreasonable errors compared to other patients. The AMRE% errors for VOIs drawn on the normal lung, brain, heart, and liver by input data sets consisting of SUV plus passes 13 to 9 were 74.3%, 55.63%, 71.77%, and 74.3% respectively. Moreover, unlike the general trend of error decreasing when adding more passes at a time, for this particular patient, the error increased by adding more passes. Due to these significant differences, this data set was considered an outlier and excluded from further evaluation. Images of this patient and additional explanations are provided in the supplementary section (Supplementary Fig. 1).

Table 1 summarizes the mean and standard deviation of the quantitative metrics. MAE, ME, MRAE%, RE%, RMSE, MSE, PSNR, and SSIM metrics were calculated between reference and predicted *K*_*i*_ images. The models trained by SUV plus passes 13 to 9 and passes 13 to 11 were depicted as representative examples of models producing the lowest errors and comparable errors with the lowest number of input passes, respectively. The metrics were calculated for all 18 patients included in the study protocol. The results demonstrate insignificant differences between the 2 suggested models.

**Table 1:** Quantitative analysis of the outcome of using two data sets as input for neural network training consisting of SUV plus passes 13 to 9 and passes 13 to 11

Parameter	SUV plus passes 13 to 9 input	Passes 13 to 11 input
MAE	1.35 × 10^−5^ ± 3.21 × 10^−6^	1.42 × 10^−5^ ± 3.39 × 10^−6^
ME	1.75 × 10^−6^ ± 7.04 × 10^−6^	1.84 × 10^−6^ ± 7.42 × 10^−6^
MRAE%	7.45 ± 0.94%	7.85 ± 0.99%
RE%	4.54 ± 2.93%	4.79 ± 3.08%
RMSE	7.58 × 10^−5^ ± 4.06 × 10^−5^	7.99 × 10^−5^ ± 4.28 × 10^−5^
MSE	7.31 × 10^−9^ ± 6.7 × 10^−9^	8.13 × 10^−9^ ± 7.44 × 10^−9^
PSNR	46.89 ± 7.66	46.5 ± 7.62
SSIM	1.00 ± 6.7 × 10^−7^	1.00 ± 7.42 × 10^−7^

**Fig. 4:**
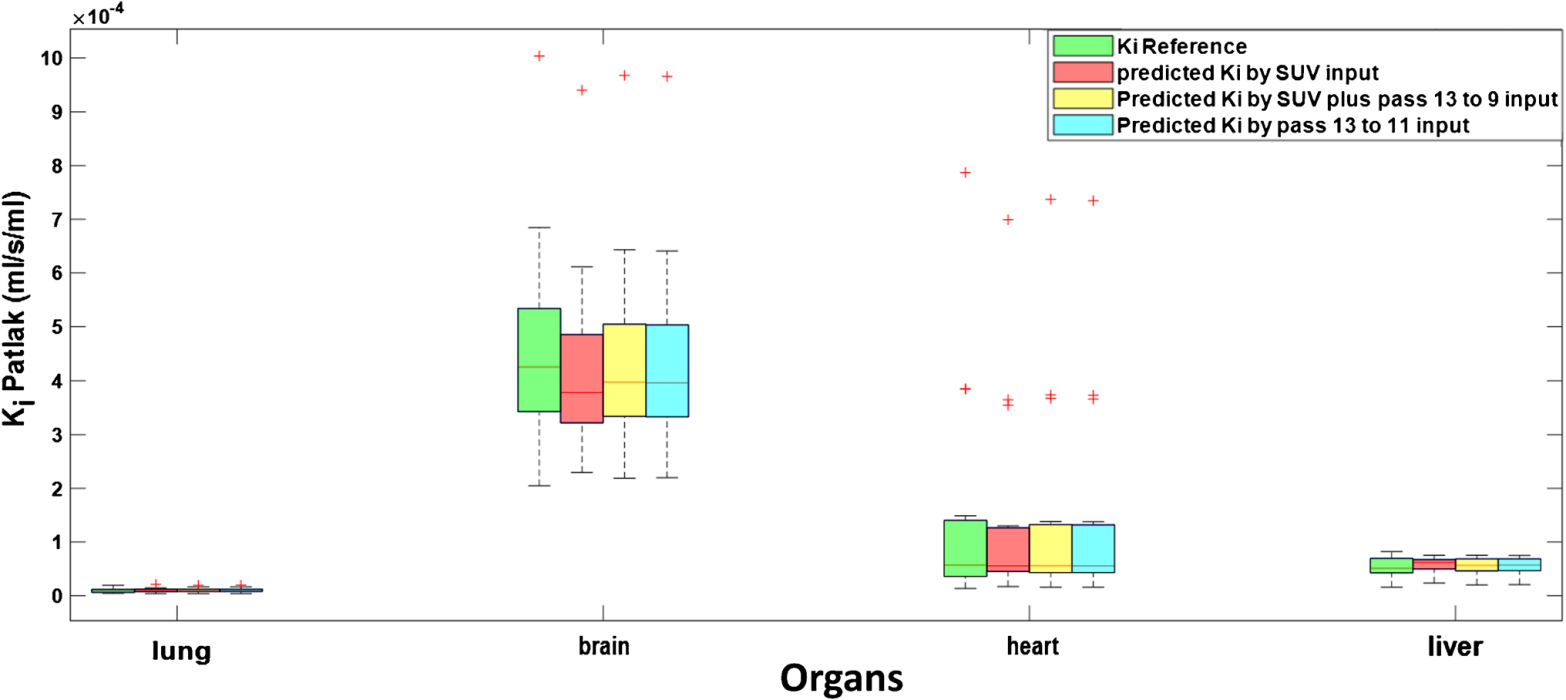
Whisker plots comparing reference and predicted *K*_*i*_-Patlak values for different organs from parametric images generated using the different models. The models trained with SUV plus passes 13 to 9 and passes 13 to 11 are in close agreement

Figure 4 shows box plots comparing reference and estimated *K*_*i*_-Patlak parameters derived using SUV alone, SUV plus passes 13 to 9, and passes 13 to 11 for the liver, lung, heart, and the brain. It can be observed that the depicted results exhibited insignificant differences between the model using SUV plus passes 13 to 9 and the model with passes 13 to 11 as input. On average, the brain (4.71 ± 2.88%), heart (9.39 ± 9.59%), liver (10.3 ± 9.62%), and lung (10.4 ± 11.15%) ranked from the lowest to the highest in terms of AMRE% metric. The predicted models resulted in lower errors in the brain and larger errors in lung and liver organs. The RE% in the lung, brain, heart, and liver organs were 2.84 ± 15.18%, − 3.84 ± 4.03%, 5.25 ± 12.5%, and 8.44 ± 11.37%, respectively. Overall, *K*_*i*_ in the brain was underestimated, whereas it was overestimated in the remaining organs by models trained with SUV plus passes 13 to 9. Moreover, the Spearman correlation coefficients and their *p* values were calculated for the liver, heart, brain, and lung organs for performing the correlation analysis. Supplementary Table 1 shows the Spearman correlation coefficients of the liver for models trained with input data of SUV images alone, SUV plus passes 13 to 9, and passes 13 to 11. All coefficients have a *p* value less than 0.01. Therefore, the results exhibited a very strong correlation with reference organ values. The mean correlation coefficients between the reference and the models predicted by SUV images alone, SUV plus passes 13 to 9, and passes 13 to 11 are about 0.96, 0.99, and 0.99, respectively. Supplementary Table 2 shows the correlation coefficients for the models trained by previously mentioned inputs and the heart organ. All *p* values were less than 0.01. Supplementary Table 3 shows the correlation coefficients for the brain. For patients number #4 and #7, the correlation coefficients were not calculated since the brain had been cropped from the image due to large movement of the head across different acquisitions for these patients. Moreover, for patient number #3, very weak or no correlation was detected for the model trained by only SUV images as input. For the rest of patients, *p* values were less than 0.01. Supplementary Table 4 shows the Spearman correlation coefficients for the lung with *p* values less than 0.01, which shows a very strong correlation between the predicted and reference images. Again, it can be seen that for SUV plus passes 13 to 9 as input data and passes 13 to 11, the correlation coefficients are very close to each other compared to the model trained by only SUV images as input.

**Fig. 5:**
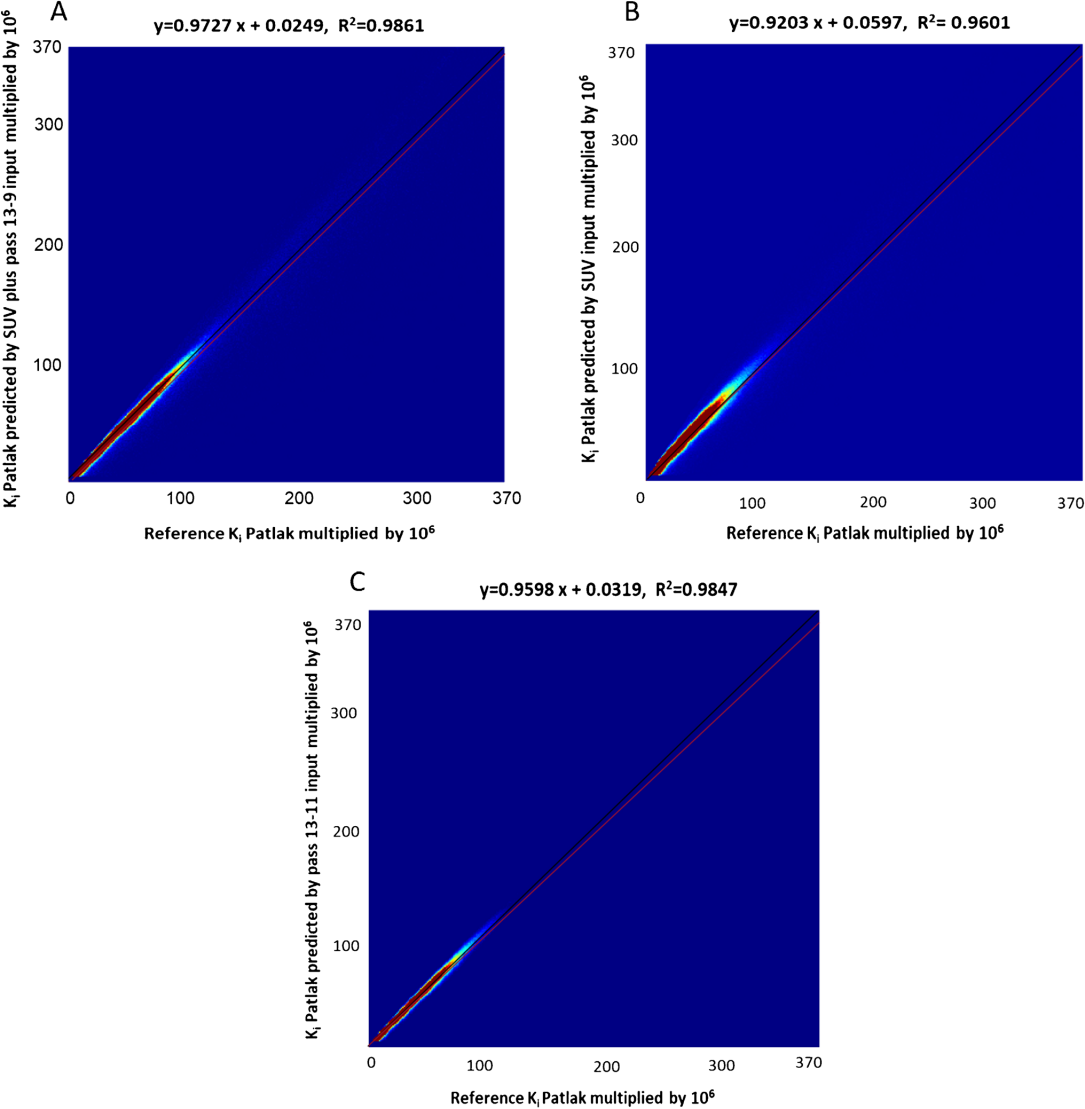
Joint histogram analysis of the voxelwise correlation between reference *K*_*i*_-Patlak maps and **A** *K*_*i*_-Patlak maps predicted by SUV plus passes 13 to 9 as input, **B** *K*_*i*_-Patlak maps predicted by only SUV, and **C** *K*_*i*_-Patlak maps predicted by passes 13–11

A voxelwise assessment of the net influx rate (*K*_*i*_-Patlak) was performed through the joint histogram analysis between reference and predicted *K*_*i*_-Patlak maps generated by the different models. Figure 5 illustrates the high correlation coefficient (0.986) and slope (0.973), respectively, obtained when using SUV plus passes 13 to 9 as input. The correlation coefficients and the slopes produced by both models were almost similar, which proves the efficacy of the simplified model using only passes 13 to 11 as input data set for model training.

### Quantitative and qualitative evaluations of documented regions of interest

**Fig. 6:**
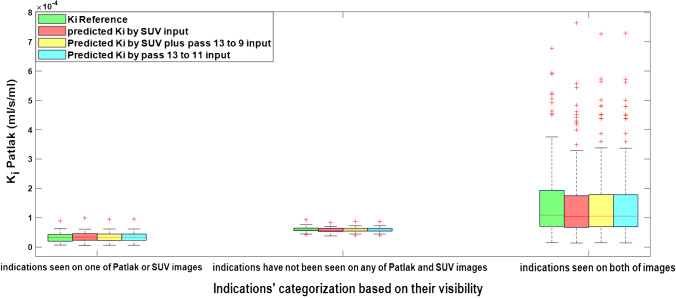
Whisker plots showing mean *K*_*i*_-Patlak values for reference and predicted *K*_i_ images by the different models for different lesion categories based on their visibility

Overall, 242 regions including primary tumors and other distant metastases, lymph nodes, and inflammatory uptakes were analyzed. When the number of indications for a specific organ or anatomical region is more than 5, the extra regions of interest were excluded in subsequent evaluations to reduce the bias. Since patient # 3 was excluded from further evaluation, a total of 221 regions were utilized for final evaluations. The type of lesion was not important in our analysis. Owing to differences in lesion detectability between SUV and *K*_*i*_-Patlak images [8], the lesions were categorized into 3 groups; (i) visible on either Patlak or SUV images, (ii) invisible on both Patlak and SUV images, and (iii) visible on both. Figure 6 shows the box plots of *K*_*i*_-Patlak for reference and predicted images with different inputs presented by different groups of indications. It can be seen that the predicted parameters are in good agreement with the reference values.

**Fig. 7:**
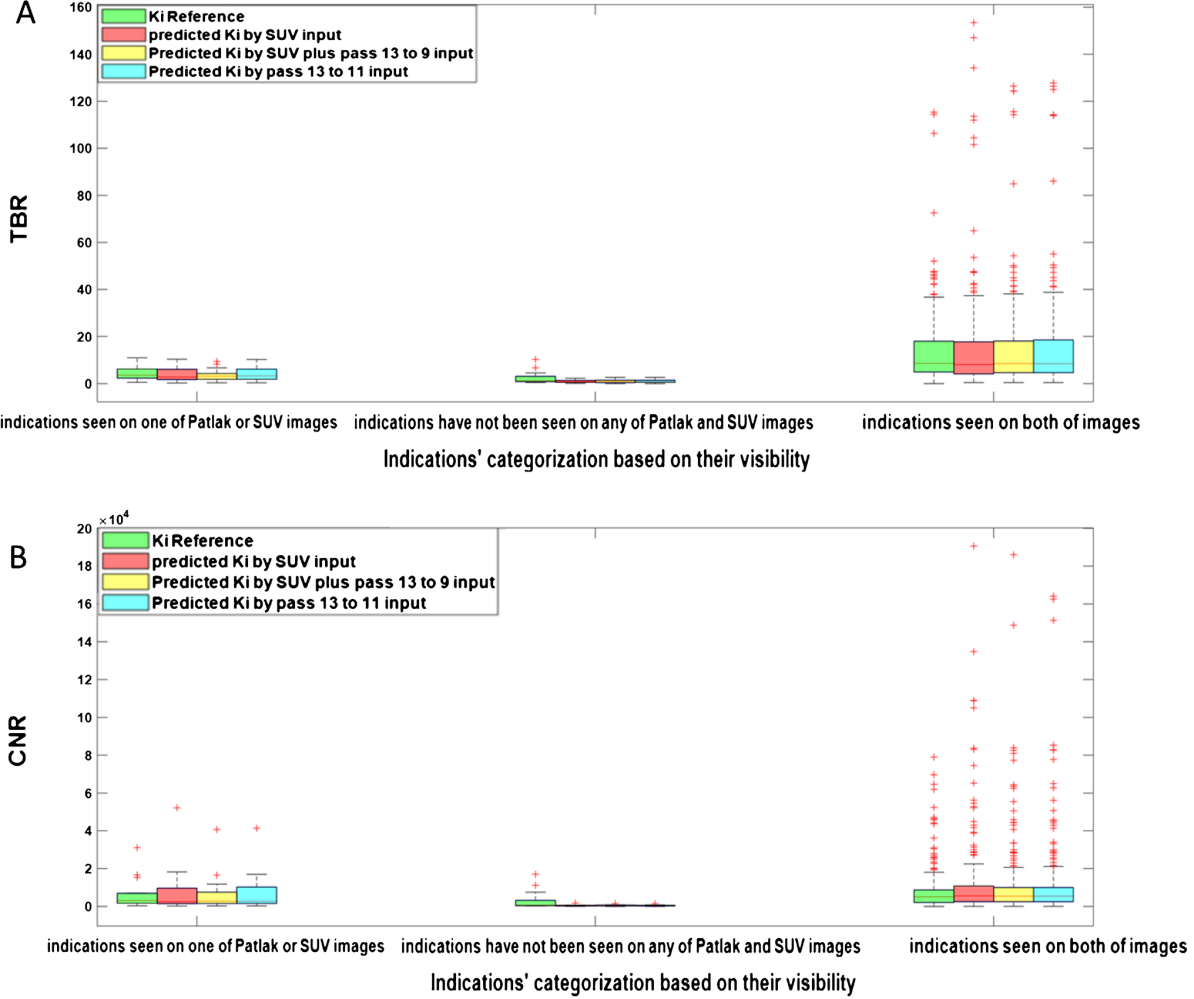
Whisker plots showing **A** TBR and **B** CNR for reference and predicted *K*_*i*_-Patlak using the different models for different lesions

Figure 7 depicts the TBR and CNR metrics for the categorized lesions calculated on reference and predicted images. The plot reveals that the predicted models underestimate the TBR and CNR indices for lesions invisible on both SUV and Patlak images. The TBR and CNR scores of lesions visible on both of Patlak and SUV images are very close to the reference values especially for models predicted using SUV plus passes 13 to 9 and passes 13 to 11 as input data sets.

**Fig. 8:**
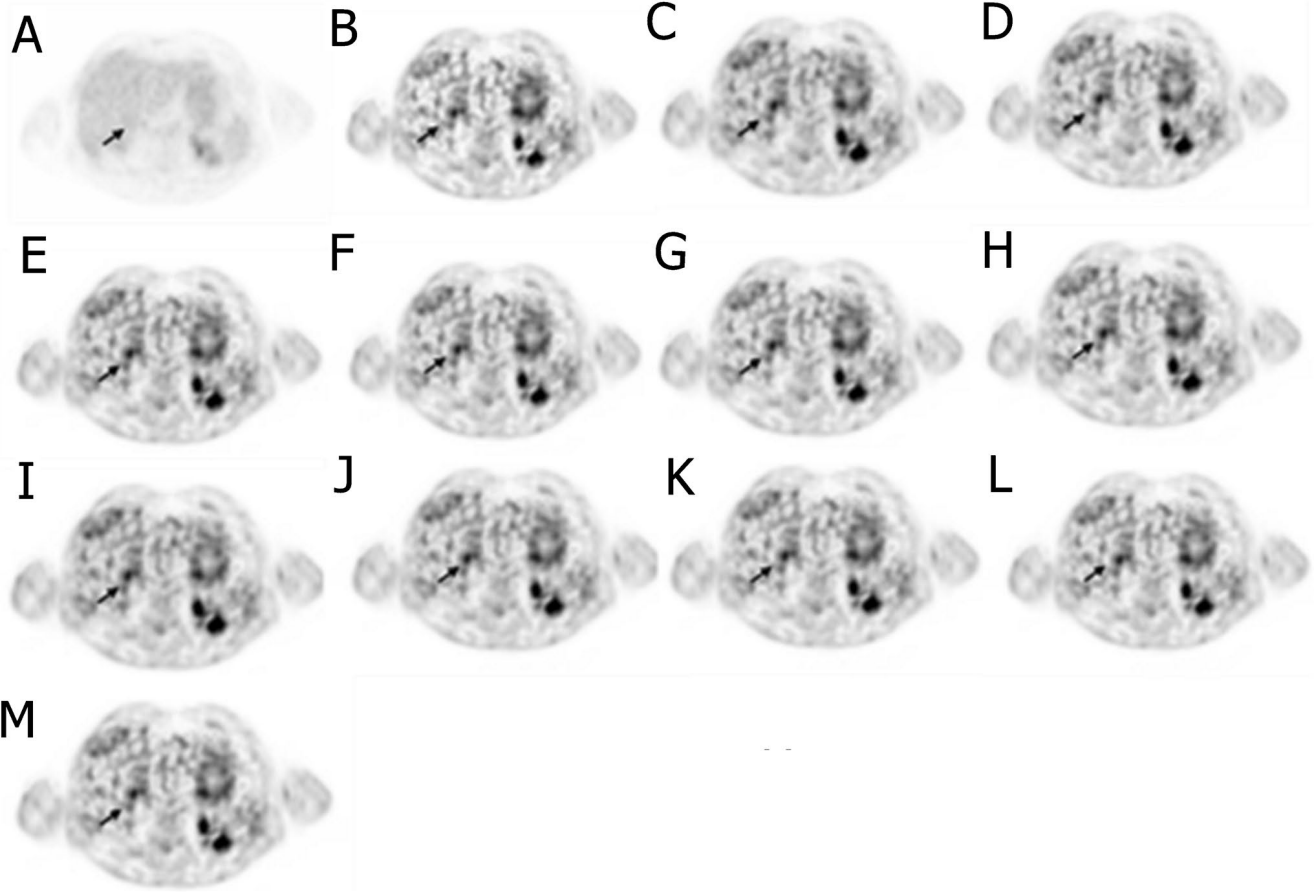
Case of a biopsy-proven carcinoma showing: **A** SUV image (the range is between 0 and 1 g/ml), **B** ground truth, and predicted *K*_i_-Patlak images by models trained using **C** SUV only, **D** SUV plus pass 13, **E** SUV plus passes 13–12, **F** SUV plus passes 13–11, **G** SUV plus passes 13–10, **H** SUV plus passes 13–9, **I** pass 13, **J** passes 13–12, **K** passes 13–11, **L** passes 13–11, and **M** passes 13–9 as input data set. The ground truth and all predicted images are within the range 0 to 0.018 ml/s/ml

**Fig. 9:**
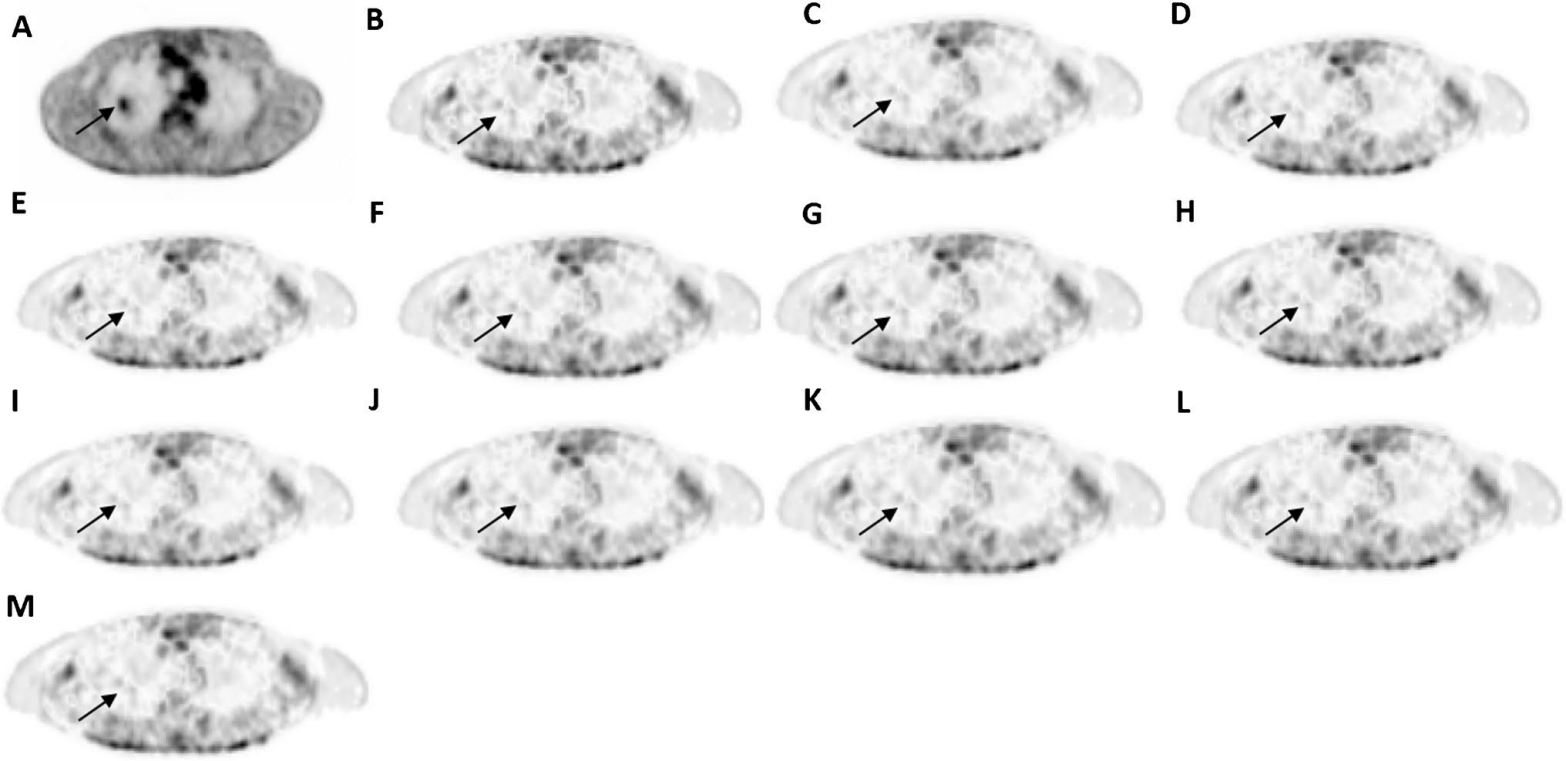
Case of an inflammatory lung lesion, initially declared as malignant (unproven) in the standard SUV report, confirmed benign by biopsy during the follow-up. **A** SUV image (the range is between 0 and 0.15 g/ml), **B** ground truth image of *K*_i_-Patlak and predicted images of *K*_i_-Patlak by input **C** just SUV, **D** SUV plus pass 13, **E** SUV plus passes 13–12, **F** SUV plus passes 13–11, **G** SUV plus passes 13–10, **H** SUV plus passes 13–9, **I** pass 13, **J** passes 13–12, **K** passes 13–11, **L** passes 13–11, **M** passes 13–9. The range of the ground truth image and all the predicted images are in the range of 0 to 0.006 ml/s/ml

Table 2 compares various metrics (AMRE%, TBR, and CNR) between the reference and predicted parameters for all categories of lesions. The lesions were categorized into 3 groups visible on either Patlak or SUV images, invisible on both Patlak and SUV images, and visible on both. For a detailed investigation of the first group and lesion detectability, this group was divided into two groups: invisible on SUV but visible on *K*_*i*_ and visible on SUV but invisible on *K*_*i*_ images. In this way, we attempted to investigate lesion detectability on the predicted images compared to the reference images. Moreover, the relative errors of TBR and CNR were also calculated. There were two liver lesions non-hypermetabolic on SUV images but hypermetabolic on *K*_*i*_-Patlak images. Both were visible on all predicted *K*_*i*_ images even on the model trained only by SUV images. Figure 8 shows a case of a biopsy-proven carcinoma described as hypervascular on the contrast-enhanced CT examination but as non-metabolic on the standard SUV images. This was reported as hypermetabolic on the reference *K*_*i*_ images; yet, biopsy later confirmed it as hepatocellular carcinoma. The AMRE% for the models trained by only SUV, SUV plus pass 13, SUV plus passes 13 to 12, SUV plus passes 13 to 11, SUV plus passes 13 to 10, SUV plus passes 13 to 9, pass 13, passes 13 to 12, passes 13 to 11, passes 13 to 10, and passes 13 to 9 were 11.78%, 9.06%, 8.22%, 6.93%, 6.76%, 6.7%, 12.18%, 8.83%, 7.07%, 6.79%, and 6.73%, respectively. The TBR_ref_ and CNR_ref_ for this lesion are 3.70 and 1611.44, respectively. They are (TBR/CNR) 2.75/1358.26, 2.93/1422.49, 2.99/1441.41, 3.09/1469.54, 3.1/1473.23, 3.11/1474.75, 2.73/1348.22, 2.95/1427.62, 3.08/1466.56, 3.1/1472.39, 3.11/1473.78, and 3.11/1611.44 for SUV only, SUV plus pass 13, SUV plus passes 13–12, SUV plus passes 13–11, SUV plus passes 13–10, SUV plus passes 13–9, pass 13, passes 13–12, passes 13–11, passes 13–11, and passes 13–9 as input data set, respectively. The TBR and CNR metrics obtained from the models trained using the input data set after adding pass 11 exhibited better agreement with reference values compared to models trained by other input data sets. The quantitative analysis showed that the outcome of the model with SUV plus passes 13 to 9 had the least error and nearest TBR and CNR to the reference values. Hence, the model trained with passes 13 to 11 could be considered the optimal model with respect to the acquisition time and quantification accuracy. In addition, the mean AMRE% of the two liver lesions was 4.2%. The mean relative error of TBR and CNR for models trained using passes 13 to 11 and SUV plus passes 13 to 9 for these 2 lesions were − 26.04% and − 18.94%, respectively, indicating underestimation of these parameters by the mentioned models. There were 14 lesions visible on SUV images but invisible on *K*_*i*_ images. Visually, all these lesions were invisible on all predicted images as well as their reference counterparts. The mean AMRE% for these lesions was 8.44 ± 6.44%, whereas the mean relative error of TBR and CNR for these lesions were − 10.44 ± 11.05% and − 0.75 ± 15.41%, respectively. Figure 9 depicts a case of an inflammatory lung lesion initially reported as malignant (unproven) in the standard SUV report, later confirmed as benign by biopsy during follow-up. This lesion was invisible on all *K*_*i*_ predicted images and had AMRE%, TBR, and CNR of 10.57%, 8.21, and 40,704.05, respectively, for SUV plus passes 13 to 9. The AMRE%, TBR, and CNR of this lesion for the model with passes 13 to 11 were 12.23%, 8.25, and 41,373.41, respectively.

**Table 2:** RMAE%, TBR, and CNR for the reference and predicted images and the mean relative errors of TBR and CNR between the reference and predicted images. The predicted images were generated by the model with SUV plus passes 13 to 9

Category of lesions	All	Invisible on SUV but visible on *K*_i_	Visible on SUV but invisible on *K*_i_	Invisible on both
Number of lesions	221	2	14	16
AMRE%	5.71 ± 7.93%	4.2 ± 3.54%	8.44 ± 6.44%	5.71 ± 3.34%
Mean reference TBR	14.09 ± 18.98	5.19 ± 2.1	4.51 ± 3.27	1.19 ± 0.88
Mean reference CNR	9621.6 ± 16,822.59	4127.23 ± 3557.86	7562.62 ± 8529.24	485.1 ± 279.39
Mean predicted TBR	13.97 ± 20.45	3.69 ± 0.81	4.17 ± 3.18	1.04 ± 0.77
Mean predicted CNR	10,886.27 ± 20,319.7	3082.43 ± 2273.6	8336.13 ± 10,847.93	506.58 ± 342.24
Mean relative error of TBR%	− 6.33 ± 13.23%	− 26.04 ± 14.27%	− 10.44 ± 11.05%	− 18.41 ± 28.6%
Mean relative error of CNR%	8.32 ± 27.1%	− 18.94 ± 14.79%	− 0.75 ± 15.41%	− 5.62 ± 31.8%

There were 16 lesions invisible on both SUV and *K*_*i*_-Patlak images. Visually, all predicted models could not depict these lesions similar to reference *K*_*i*_-Patlak slopes. The mean AMRE%, TBR_ref_, and CNR_ref_ for these lesions were 5.71 ± 3.34%, 1.19 ± 0.88, and 485.1 ± 279.39, respectively. The mean predicted TBR and CNR for the model with SUV plus passes 13 to 9 were 1.04 ± 0.77 and 506.58 ± 342.24, respectively. Furthermore, it can be inferred from Table 2 that our models tend to underestimate the TBR and CNR parameters in all categories.

## Discussion

The results of this study indicated that by incorporating a small number of passes (13 to 11) and applying a deep learning model, Patlak parameter (*K*_*i*_) can be estimated without the use of an input function and the traditional model fitting approach. The visual inspection of Figs. 2, 8, and 9 revealed that the parametric maps estimated by the different models were in good agreement with the tracer kinetics model generated (reference) maps. The predicted maps (Figs. 8 and 9) reproduced relatively well the lesion detectability performance of *K*_*i*_ maps compared to their reference counterparts.

Techniques enabling to generate Patlak maps with only two passes provided the PIF is known [11]. The major limitation was that the patients should be positioned in the scanner twice for two separate acquisitions. This method required a delay of 40–60 min between frames and an acquisition time of 65–85 min, which is inconvenient for the patient. Moreover, due to the time difference between the frames, co-registration is also needed for image alignment, not to mention the need for an IF. Our DL-based technique proposed as few as 3 passes without any time interval between passes for acquiring *K*_*i*_ maps. Karakatsanis et al. [7] suggested 6 whole-body passes with a constant time frame of 45 s for each bed position as the optimal acquisition protocol. Our proposed technique seemed to indicate that three passes were sufficient to produce acceptable results.

As elaborated in the previous sections, extracting the input function has its own difficulties and is one of the obstacles to introducing dynamic PET imaging in clinical routine. Moreover, the optimization of this method by reducing the acquisition time is an urgent step for its feasibility in the clinic due to organ movement or body motion during long acquisition times. The model proposed in this work could overcome these difficulties using deep learning pipelines.

One of the advantages of dynamic WB imaging is its ability to remove the background uptake, which enables to highlight small and less FDG avid tumors especially located in the liver [8], a feature that was preserved by our deep learning-based approach (Fig. 8) even when using a single SUV image as input. In normal parameter estimation by linear regression, voxelwise mapping is performed between the input and output images using the regression formula. In contrast, the deep learning-based models benefit from the holistic view advantage of taking into account a number of training examples and voxels of other tissue types and not only information of the same voxel [37]. This feature can reduce the adverse impact of noisy voxels by incorporating the information from voxels residing in the same tissue types (Figs. 8 and 9).

In this work, the deep learning models were trained in 2D fashion. However, 3D implementation of these models considering multiple slices as input might improve the quality of the predicted parametric images in terms of continuity across the slices. Nevertheless, the implementation of 3D networks is challenging in terms of computational burden, particularly for scenarios involving 4 or 5 input PET images. Regarding the networks trained with 4 or 5 input channels in this study, the implementation of 3D models was not practically feasible. In the case of 3D models, we had to use a batch size of 1, which led to suboptimal performance. In this regard, considering previous works comparing 2D and 3D CNNs, the improvement brought by 3D implementations was not significant [45, 46]. For example, Seo et al. [45] proposed a quasi-3D U-Net architecture with an input channel taking three consecutive slices. Their results indicated no significant differences between the performance of the quasi-3D U-Net and 2D U-Net approaches. Moreover, Son et al. [46] compared the diagnostic performance of 2D CNNs vs 3D CNNs on a clinical validation and test data sets to score the slice-level amyloid positivity. They reported that the 2D-based approach was not inferior to the 3D-based approach for their specific task considering the fact that human readers score amyloid positivity in a slice-by-slice manner. Figures 4, 5, 6, and 7 prove the close proximity of predicted *K*_*i*_-Patlak images generated by the different models using SUV alone and SUV plus passes 13 to 9 and passes 13 to 11. The inspection of the results revealed that the number of required input data sets can be determined depending on the application. For example, if the target of dynamic imaging is lesion detectability, training the models with only SUV images could be sufficient to synthesize parametric images. Alternatively, if quantitative analysis is sought, the number of inputs should be increased to 3 passes. Figure 6 indicates that the visibility or invisibility of lesions were not affected by K_*i*_-Patlak values, although the TBR and CNR metrics were affected by lesions invisible on both SUV and *K*_*i*_-Patlak images (Fig. 7). The invisible group of lesions on predicted images, including models with only SUV and SUV plus passes 13 to 9 and passes 13 to 11 as input was underestimated by TBR and CNR factors. Two groups “with SUV” and “without SUV” were implemented for model training to investigate the impact of SUV compared to the time-series images (passes). The role of SUV in model performance was equivalent to that of a pass as acceptable results were achieved even with models trained “without SUV” group (Fig. 3).

The assessment of the impact of incorporating the input functions warrants further investigation using a larger cohort and different acquisition protocols with and without using the input function information. Although it may be considered an advantage of our model, it should be noted that the data used in this study had fixed temporal resolution and acquisition parameters. The subject-specific AIF of our data set had small discrepancies in the peak magnitude and peak time points. However, the trained network could intrinsically learn the relationship between the AIF and the intended parameters through the end-to-end mapping of input and output of the network and the applied loss function taking into account the encompassed tracer kinetics model [37]. Therefore, new models may need to be examined for other tracers/acquisition protocols.

Kotasidis et al. [23] simulated an eight-frame sliding window to investigate the optimum Patlak regression/acquisition window. Their results indicated that for windows starting at the 1st CBM pass, a positive bias was formed owing to the lack of linearity at early time frames. They suggested using data after the 5th CBM pass. Since the same protocol was used in this work, it was decided to start from the last pass for model training to avoid the linear region in the first frames. We propose the utilization of intermediate passes (starting from pass 5) to investigate and compare the impact of other time frame windows on the model predictions. This work reported the results starting from the last pass, since our objective was to demonstrate the feasibility of deep learning methods in generating direct Patlak parameters.

It is worth mentioning that the accuracy of our method depends on the accuracy of the chosen reference output. The standard Patlak analysis is based on the assumption of irreversible kinetics (*k*_4_ ~ 0), which is not a completely correct assumption especially in the liver for ^18^F-FDG [21]. The use of predetermined *k*_4_ values [36] and generalized Patlak model [3] are among the proposed solutions for tackling this issue. Since our objective was to investigate the applicability of this DL-based approach, standard Patlak analysis was implemented to generate the reference *K*_*i*_-Patlak maps. The same models could be retrained with reference data sets generated with more complex models. The major limitation of our study was the small sample size (19 patients) with studies acquired using a hybrid protocol [22].

## Conclusion

This study demonstrated the feasibility of DL-based direct inference of the pharmacokinetic *K*_*i*_-Patlak parameters. The qualitative and quantitative analysis revealed comparable results to the standard of reference. Considering the lesion detection capability of the proposed model, the model would be able to depict the lesions visible on *K*_*i*_-Patlak images by using only SUV images as input. Further assessment and validation using a larger sample size covering a variety of clinical indications is guaranteed.

## Supplementary Information

Below is the link to the electronic supplementary material.

Supplementary file1 (PDF 598 KB)
