# Supplementary material for: Direct inference of Patlak parametric images in whole-body PET/CT imaging using convolutional neural networks
Source: Eur J Nucl Med Mol Imaging. 2022 Jun 18;49(12):4048–63. doi: 10.1007/s00259-022-05867-w (PMC9525418; doi:10.1007/s00259-022-05867-w)
Supplement: Supplementary file 1 — Supplementary file1 (PDF 598 KB) [file 259_2022_5867_MOESM1_ESM.pdf]

Supplementary Tables

Supplementary Table 1. The Spearman correlation coefficients of the liver calculated between reference and predicted images by models trained with SUV images alone, SUV plus passes 13 to 9 and passes 13 to 11. The p-values are less than 0.01.

Patient number	SUV input alone	SUV plus passes13 to 9 input	Passes 13 to 11 input
**1**	0.942	0.982	0.980
**2**	0.970	0.992	0.991
**3**	0.960	0.971	0.971
**4**	0.980	0.995	0.995
**5**	0.983	0.983	0.981
**6**	0.959	0.988	0.987
**7**	0.960	0.989	0.988
**8**	0.981	0.995	0.994
**9**	0.978	0.994	0.993
**10**	0.983	0.996	0.995
**11**	0.984	0.996	0.995
**12**	0.953	0.987	0.985
**13**	0.970	0.992	0.991
**14**	0.963	0.990	0.988
**15**	0.969	0.992	0.991
**16**	0.877	0.961	0.956
**17**	0.966	0.991	0.990
**18**	0.986	0.997	0.996
**19**	0.956	0.992	0.991
**Average**	0.964±0.024	0.989±0.009	0.987±0.01

Supplementary Table 2. The Spearman correlation coefficients of the heart calculated between reference and predicted images by models trained with SUV images alone, SUV plus passes 13 to 9 and passes 13 to 11. The p-values are less than 0.01.

Patient number	SUV input alone	SUV plus passes 13 to 9 input	Passes 13 to 11 input
**1**	0.970	0.992	0.991
**2**	0.991	0.997	0.997
**3**	0.865	0.843	0.845
**4**	0.994	0.998	0.998
**5**	0.892	0.967	0.963
**6**	0.982	0.995	0.994
**7**	0.906	0.974	0.971
**8**	0.962	0.990	0.989
**9**	0.984	0.996	0.996
**10**	0.977	0.994	0.993
**11**	0.985	0.996	0.996
**12**	0.995	0.999	0.998
**13**	0.946	0.987	0.985
**14**	0.993	0.998	0.998
**15**	0.964	0.990	0.989
**16**	0.783	0.936	0.928
**17**	0.974	0.994	0.993
**18**	0.939	0.983	0.981
**19**	0.994	0.998	0.998
**Average**	0.952±0.055	0.980±0.037	0.979±0.036

Supplementary Table 3. The Spearman correlation coefficient calculated for the brain between reference and predicted images by models trained with SUV alone, SUV plus passes 13 to 9 and passes 13 to 11. Since the head was cropped from the image due to large movement of the head across different acquisitions for patient number #4 and #7, the correlation coefficients were not calculated for these patients. Moreover, since patient number #3 was an outlier, the correlation values were negative (in red and bold).

Patient's number	SUV input alone	SUV plus passes 13 to 9 input	Passes 13 to 11 input
**1**	0.991	0.996	0.996
**2**	0.991	0.997	0.996
**3**	**-0.012**	**-0.130**	**-0.121**
**4**	-	-	-
**5**	0.981	0.994	0.993
**6**	0.986	0.995	0.994
**7**	-	-	-
**8**	0.981	0.995	0.994
**9**	0.988	0.996	0.996
**10**	0.993	0.998	0.998
**11**	0.993	0.998	0.997
**12**	0.990	0.997	0.996
**13**	0.979	0.994	0.993
**14**	0.995	0.999	0.998
**15**	0.995	0.998	0.998
**16**	0.940	0.978	0.976
**17**	0.978	0.993	0.992
**18**	0.991	0.997	0.997
**19**	0.972	0.991	0.990
**Average**	0.984±0.013	0.995±0.005	0.994±0.005

Supplementary Table 4. The Spearman correlation coefficient calculated for the lung between reference and predicted images by models trained with SUV alone, SUV plus passes 13 to 9 and passes 13 to 11. The p-values were less than 0.01, demonstrating very strong correlation especially for the model predicted using as input SUV plus passes 13 to 9 and the model predicted by passes 13 to 11 which has very close values to the model predicted by SUV plus passes 13 to 9 as input data.

Patient's number	SUV input alone	SUV plus passes 13 to 9 input	Passes 13 to 11 input
**1**	0.886	0.965	0.961
**2**	0.854	0.957	0.951
**3**	0.885	0.889	0.890
**4**	0.948	0.986	0.984
**5**	0.878	0.971	0.967
**6**	0.835	0.939	0.933
**7**	0.807	0.932	0.925
**8**	0.950	0.987	0.985
**9**	0.926	0.980	0.978
**10**	0.954	0.987	0.985
**11**	0.955	0.987	0.986
**12**	0.903	0.971	0.967
**13**	0.879	0.965	0.961
**14**	0.938	0.980	0.978
**15**	0.815	0.934	0.927
**16**	0.682	0.871	0.858
**17**	0.904	0.974	0.971
**18**	0.948	0.986	0.985
**19**	0.896	0.969	0.965
**Average**	0.886±0.066	0.959±0.032	0.956±0.034

Supplementary figures

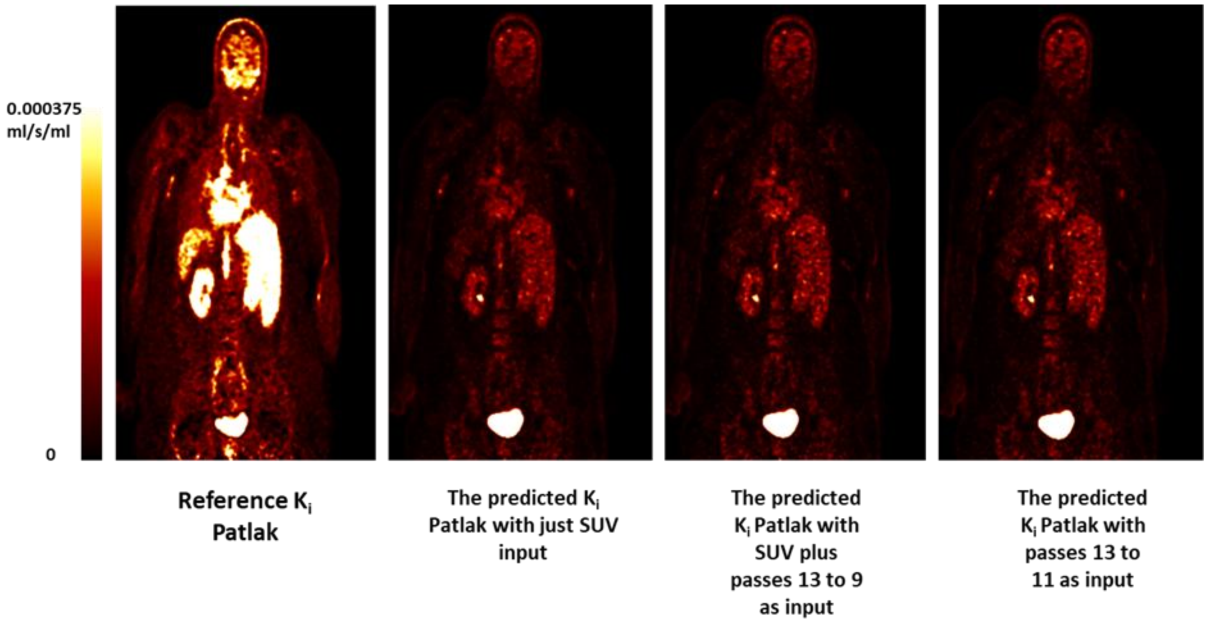

Figure 1. Representative coronal views of patient number #3 (outlier) showing the reference and predicted images derived from SUV images alone, SUV plus passes 13 to 9 and passes13 to 11 as input data.

The input function of this patient did not have any significant difference with other patients. The acquisition protocol was also the same. The only major difference was that this patient was obese (weight = 116 kg, height =162 cm) with a BMI of 44.2. However, the average BMI of other patients was 23.89±3.4. We think that the reason for the failure/suboptimal performance of the model in predicting this patient's parametric maps was the lack of such abnormal patients in the training dataset. Images of the reference K_i Patlak, the predicted images using as input SUV images, SUV plus passes 13 to 9, and passes13 to 11 are depicted in Figure 1.

The different whole-body parameters for this patient and for the predicted images using SUV images plus passes 13 to 9 as input are as follows: MAE: 1.09×10^{-5}, MRAE%: 8.75%, ME: 2.53×10^{-6}, %RE: 2.79%, RMSE: 2.29×10^{-5}, MSE: 5.26×10^{-10}, PSNR: 40.4 and SSIM: 0.9982. The average of these values for other patients, except patient number #3 are as follows: MAE: $1.36\times10^{-5}\pm3.24\times10^{-6}$, %MRAE: 7.38%±0.91%, ME: $1.7\times10^{-6}\pm7.24\times10^{-6}$, %RE: 4.64%±2.98%, RMSE: $7.88\times10^{-5}\pm3.97\times10^{-5}$, MSE: $7.69\times10^{-9}\pm6.68\times10^{-9}$, PSNR: 47.25±7.71 and SSIM: $0.9995\pm6.17\times10^{-4}$. It can be argued that the indices of this patient are not very different from the average of other patients. However, if we look into organ-based results for this patient for the predicted images using SUV images plus passes 13 to 9 as input, the absolute mean error (MAE) for the lung, liver, heart, and brain organs are 8.45×10^{-5}, 3.52×10^{-4}, 2.78×10^{-4} and 9.74×10^{-5}, respectively. The absolute mean relative error (AMRE%) for the predicted images using as input data SUV images plus passes 13 to 9 for the lung, liver, heart, and brain are 74.3%, 74.3%, 71.77%, and 55.63%, respectively. The average AMRE% for the rest of the patients, except patient number #3, for the model trained with SUV images plus passes 13 to 9 is 4.71%±2.79%, 9.39%±9.32%, 10.3%±9.35% and 10.4%±11.15% for the brain, heart, liver, and lung, respectively.

Another difference between this patient and the other patients was the trend of error decrease which is different from other patients. As it can be seen, the error in figure 3 decreases with adding passes at a time and after pass 11, the amount of error decrease is insignificant. This trend is not always true for

patient number #3. For instance, for the lung and heart, although the error decreases, the rate of error reduction is insignificant and for the brain, the AMRE% increased. The AMRE% obtained from the model trained with SUV images alone, SUV plus passes 13, SUV plus passes 13 to 12, SUV plus passes 13 to 11, SUV plus passes 13 to 10 and SUV plus passes 13 to 9 were 74.34%, 74.32%, 74.31%, 74.3%, 74.3%, 74.3%, respectively. The AMRE% for the "Without SUV" group and for the lung ROI defined on the predicted images using pass 13, passes 13 to 12, passes 13 to 11, passes 13 to 10, and passes 13 to 9 as input were 74.34%, 74.32%, 74.3%, 74.3%, 74.3%, respectively. For the brain and for the "With SUV" group, the AMRE% for the ROI defined on the predicted images using as input SUV images alone, SUV plus pass 13, SUV plus passes 13 to 12, SUV plus passes 13 to 11, SUV plus passes 13 to 10 and SUV plus passes 13 to 9 were 53.8%, 54.78%, 55.08%, 55.55%, 55.61%, and 55.63%, respectively. For the "Without SUV" group, the AMRE% for the brain ROI defined on the predicted images using as input pass 13, passes 13 to 12, passes 13 to 11, passes 13 to 10, and passes 13 to 9 were 53.65%, 54.86%, 55.5%, 55.6%, and 55.62%, respectively. It can be observed that the AMRE% increased instead as opposed to other patients. Moreover, 4 malignant lesions were detected for this patient which had large errors and the trend of decreasing error with adding passes was not observed in this case, except for one of them. As an example, there was a right bilateral axillary hypermetabolic subcutaneous thickening with AMRE% of 54.14%, 57.15%, 58.08%, 59.5%, 59.69%, and 59.75% for an ROI defined on the predicted images trained by inputs of SUV alone, SUV plus pass 13, SUV plus passes 13 to 12, SUV plus passes 13 to 11, SUV plus passes 13 to 10 and SUV plus passes 13 to 9, respectively. The AMRE% for this defined ROI for pass 13, passes 13 to 12, passes 13 to 11, passes 13 to 10 and passes 13 to 9 were 53.69%, 57.4%, 59.35%, 59.65%, and 59.72%, respectively. The error increases instead of decreasing in contrast to the common trend observed in other patients.

Since our training was performed in a 9-fold cross-validation scheme, each time, 2 datasets were excluded/regarded as external test dataset and 5% of the data were used for validation within the training.

As stated earlier, we used the entire data as external test set once. In the following, the training and validation losses obtained within the training are plotted for the different validation folds.

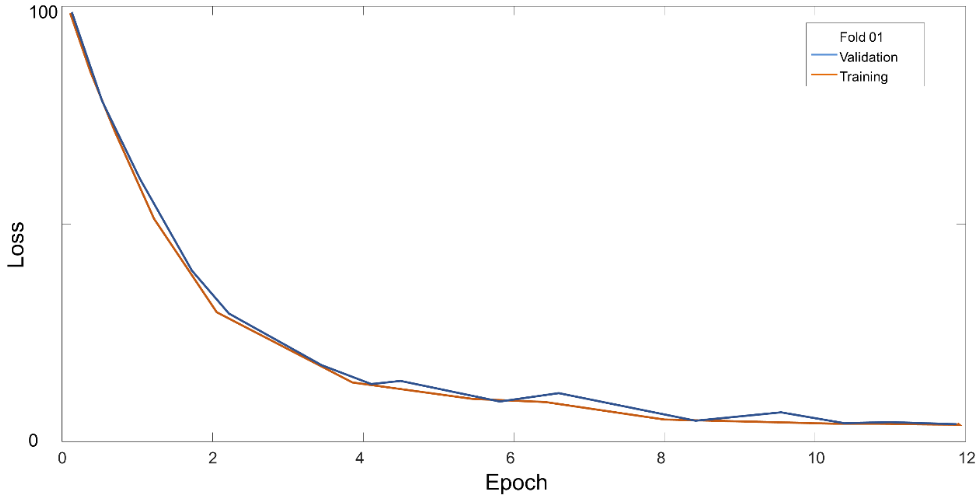

Figure 2. The training and validation losses obtained for the first fold validation.

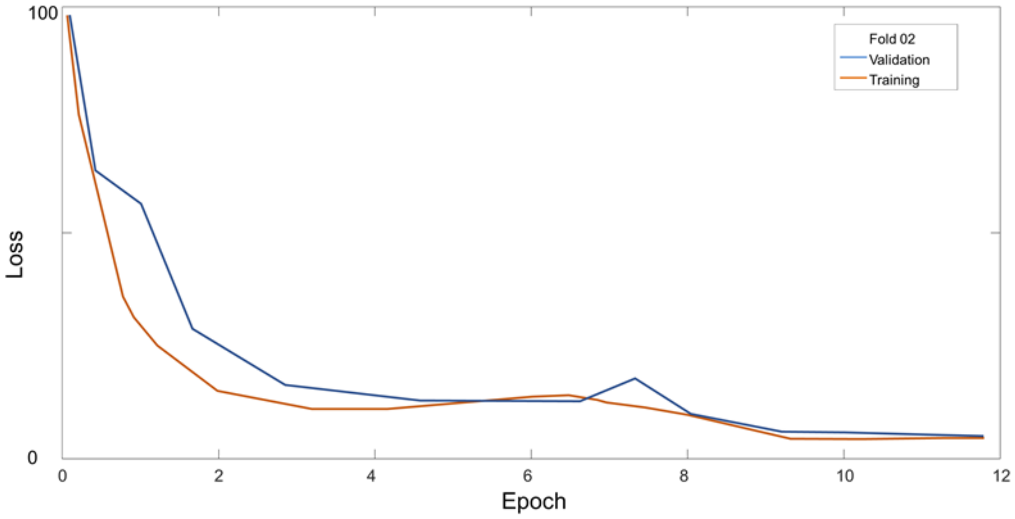

Figure 3. The training and validation losses obtained for the second fold validation.

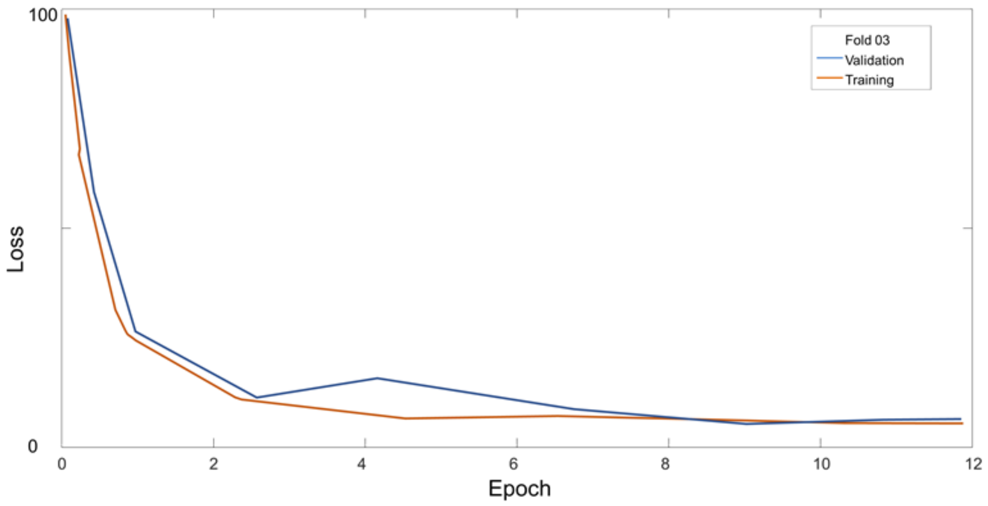

Figure 4. The training and validation losses obtained for the third fold validation.

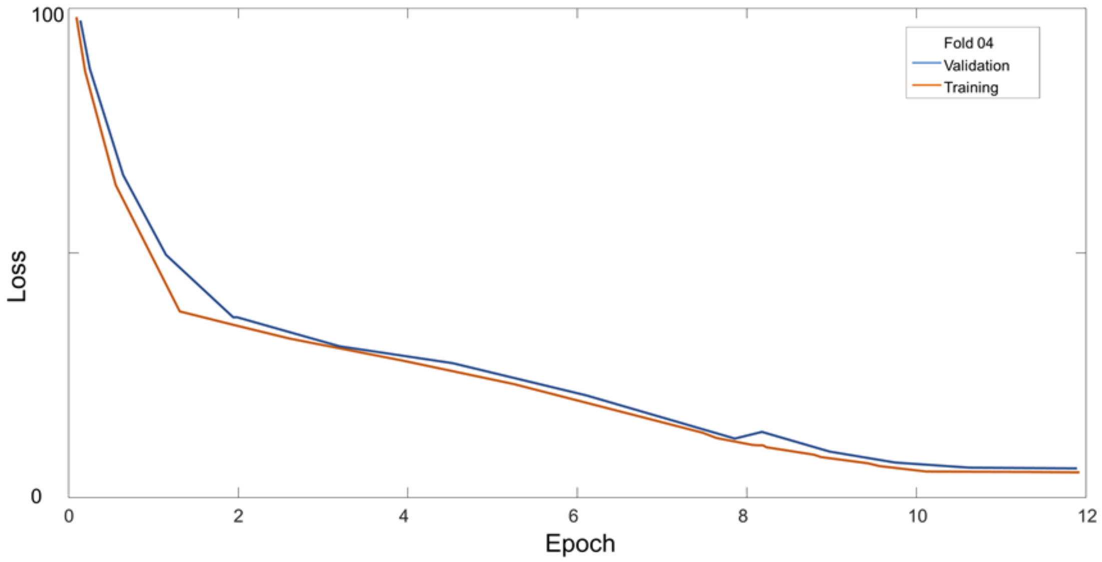

Figure 5. The training and validation losses obtained for the fourth fold validation.

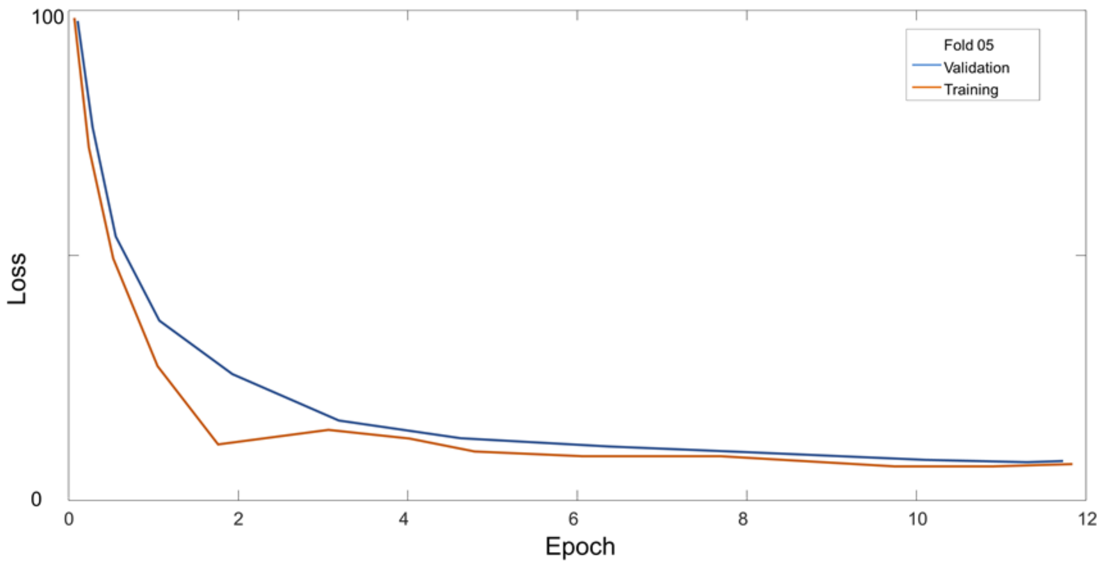

Figure 6. The training and validation losses obtained for the fifth fold validation.

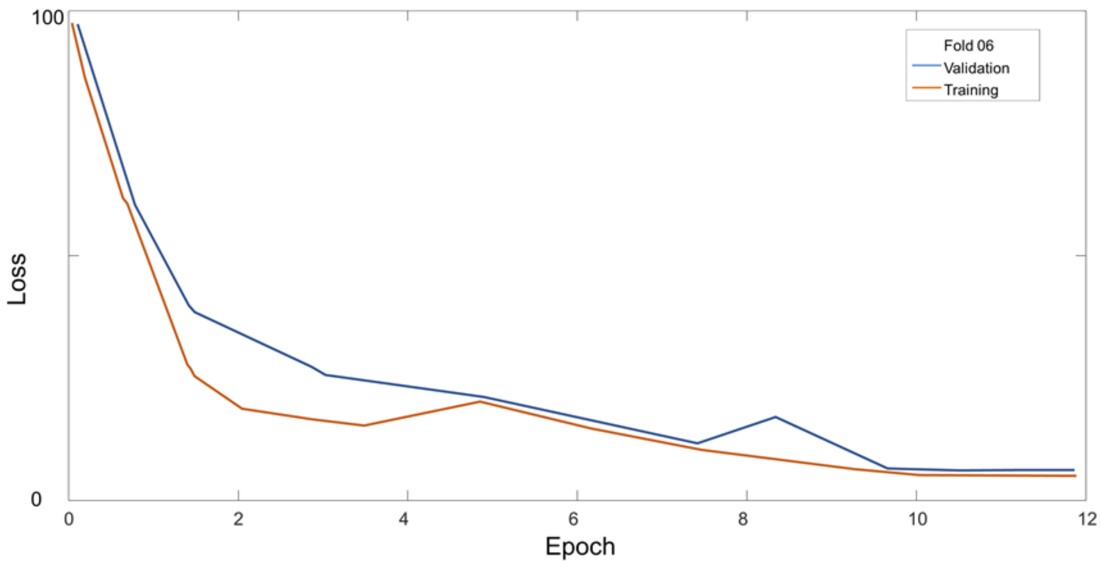

Figure 7. The training and validation losses obtained for the sixth fold validation.

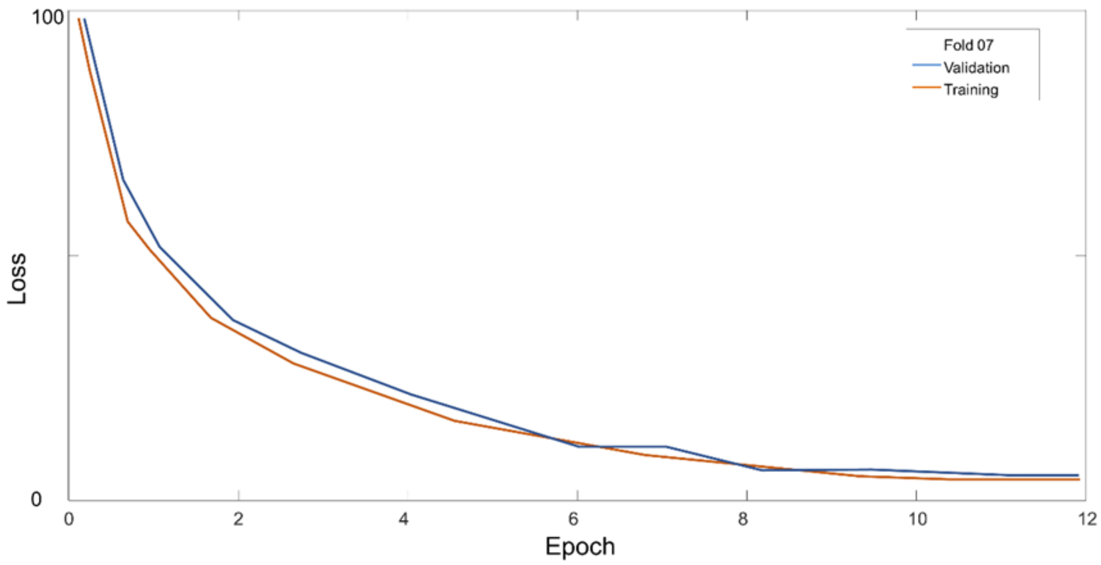

Figure 8. The training and validation losses obtained for the seventh fold validation.

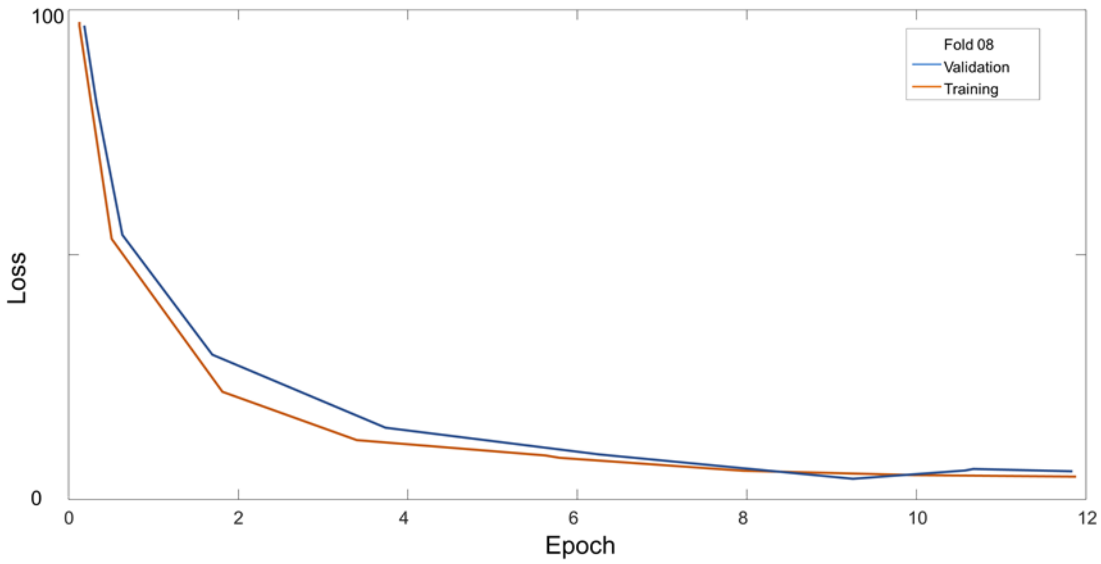

Figure 9 . The training and validation losses obtained for the eighth fold validation.

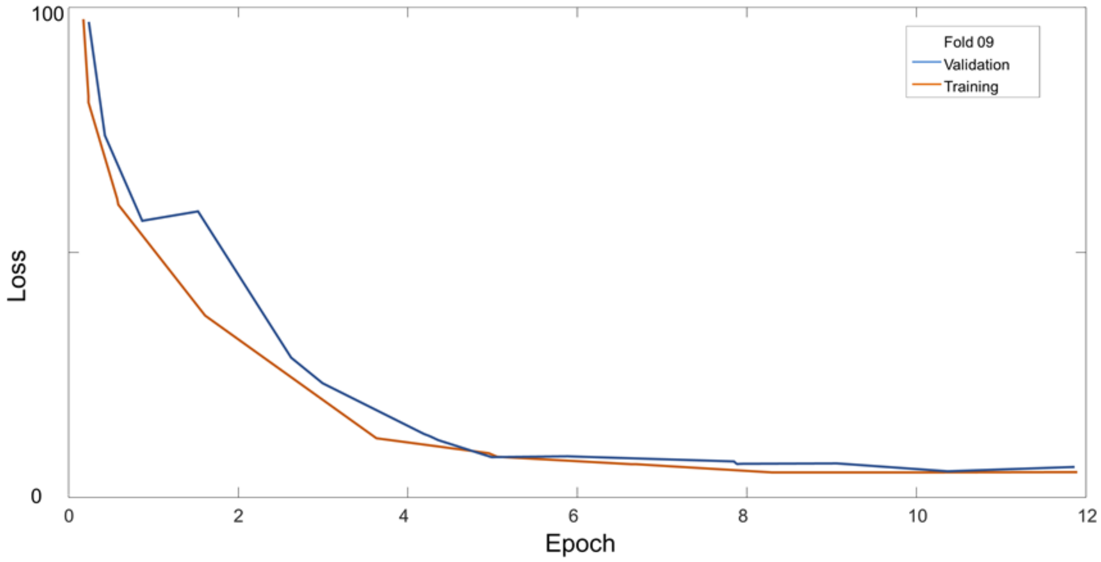

Figure 10. The training and validation losses obtained for the ninth fold validation.
